# P-1563. Assessing the Global Economic Burden of Complicated Urinary Tract Infections

**DOI:** 10.1093/ofid/ofae631.1730

**Published:** 2025-01-29

**Authors:** Edward I Broughton, Meryem Bektas, Ann Colosia, Kristi Kuper, Maria Fernandez, Amer Al-Taie, Ramy El Mahdy Kotb

**Affiliations:** Pfizer, Inc., New York, New York; RTI Health Solutions, Durham, North Carolina; RTI Health Solutions, Durham, North Carolina; Pfizer, Katy, Texas; Former Pfizer employee, NY, New York; former Pfizer employee, Epsom, England, United Kingdom; Pfizer, Katy, Texas

## Abstract

**Background:**

Complicated urinary tract infections (cUTIs) arise in patients with structural or functional abnormalities of the genitourinary tract or in those with nonurogenital comorbidities. cUTIs are common in older or catheterized persons and can be associated with high healthcare resource utilization (HCRU). We conducted a systematic literature review to understand the economic burden of cUTI.

**Figure:**
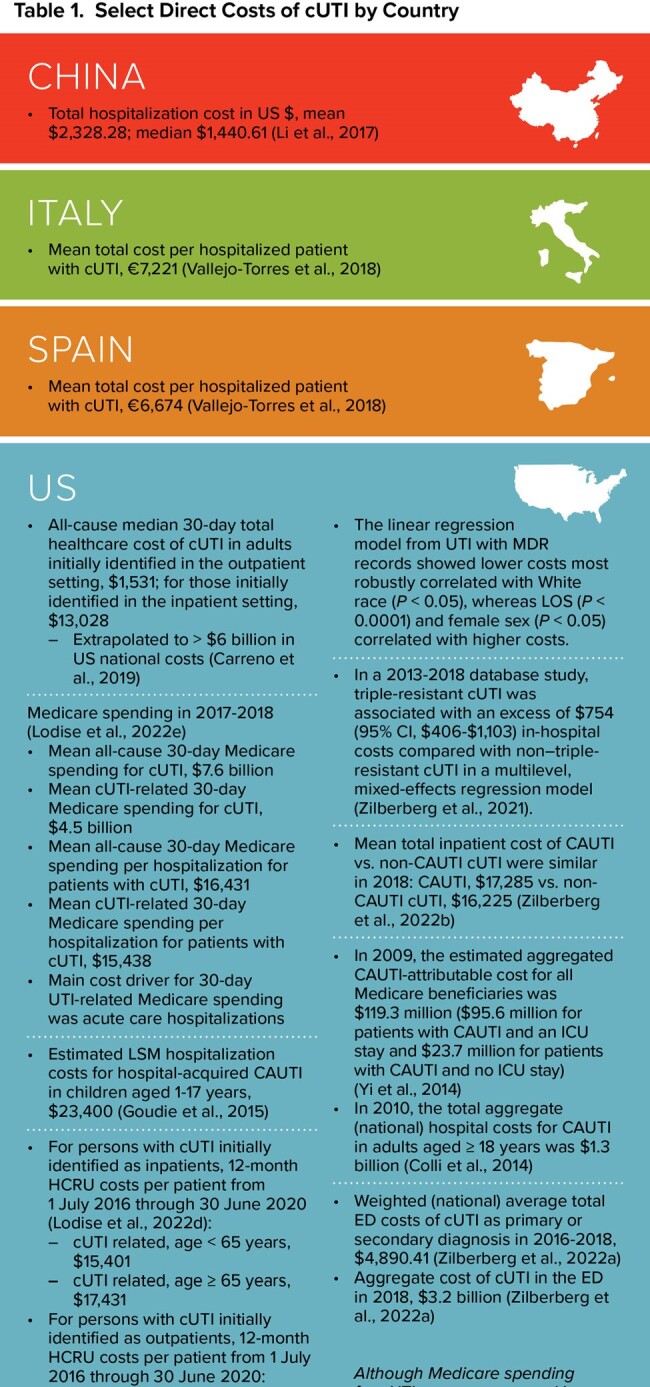

**Methods:**

A systematic literature review was conducted using PubMed, Embase, Cochrane, and EconLit databases to identify observational studies evaluating the burden of cUTI, including acute pyelonephritis and catheter-associated UTI (CAUTI), within the last decade from China, Europe, France, Italy, Germany, Japan, Spain, United Kingdom (UK), and United States (US) (PROSPERO-CRD42023454794).

**Results:**

Of 1,041 studies identified, 154 from databases were selected for full-text review; a total of 53 met the economic inclusion criteria, reporting direct costs (18 US, 2 China, 2 Spain, 1 Italy) and/or healthcare utilization (28 US, 13 Spain, 4 China, 4 Italy, 3 multicountry/European region, 2 France, 1 UK). No studies of direct cUTI costs were identified for France, Germany, Japan, the UK, or Europe.

Mean hospitalization costs per cUTI varied by country, from $2,328 in China to $23,400 for hospital-acquired CAUTI in US children. The highest costs were observed in patients with multi-drug-resistant infections. US national all-cause median 30-day total healthcare costs of cUTIs were estimated at > $6 billion (Table 1). No studies reporting the indirect cost of cUTI were identified, and costs associated with readmissions were reported in only 1 study.

Length of stay (LOS) was the most common HCRU outcome reported, and median LOS was similar among studies (7 days). Patients with CAUTI had longer LOS than controls. ICU admissions in patients with extended-spectrum β-lactamase (ESBL)–positive cUTI ranged between 3% and 27.4%.

**Conclusion:**

Available data indicate that the economic burden associated with cUTI is substantial. However, except for the US, direct costs were missing or reported in only 1 or 2 studies for the countries examined. Similarly, HCRU outcomes were missing or reported in only a few studies, except for the US and Spain.

**Disclosures:**

**Edward I. Broughton, PhD**, Pfizer: Ownership Interest **Kristi Kuper, PharmD, BCPS, FIDSA**, Pfizer: Employee|Pfizer: Stocks/Bonds (Public Company) **Maria Fernandez, PhD, MBA**, Pfizer: Stocks/Bonds (Public Company) **Amer Al-Taie, MSc**, Pfizer: Stocks/Bonds (Public Company) **Ramy El Mahdy Kotb, MD**, Pfizer: Stocks/Bonds (Public Company)

